# The Role of Reproductive Hormones in Sex Differences in Sleep Homeostasis and Arousal Response in Mice

**DOI:** 10.3389/fnins.2021.739236

**Published:** 2021-09-21

**Authors:** Jinhwan Choi, Staci J. Kim, Tomoyuki Fujiyama, Chika Miyoshi, Minjeong Park, Haruka Suzuki-Abe, Masashi Yanagisawa, Hiromasa Funato

**Affiliations:** ^1^International Institute for Integrative Sleep Medicine, University of Tsukuba, Tsukuba, Japan; ^2^Department of Molecular Genetics, University of Texas Southwestern Medical Center, Dallas, TX, United States; ^3^Life Science Center for Survival Dynamics, Tsukuba Advanced Research Alliance, University of Tsukuba, Tsukuba, Japan; ^4^Department of Anatomy, Faculty of Medicine, Toho University, Tokyo, Japan

**Keywords:** sex difference, gonadal hormone, sleep homeostasis, sleep deprivation, arousal, mice

## Abstract

There are various sex differences in sleep/wake behaviors in mice. However, it is unclear whether there are sex differences in sleep homeostasis and arousal responses and whether gonadal hormones are involved in these sex differences. Here, we examined sleep/wake behaviors under baseline condition, after sleep deprivation by gentle handling, and arousal responses to repeated cage changes in male and female C57BL/6 mice that are hormonally intact, gonadectomized, or gonadectomized with hormone supplementation. Compared to males, females had longer wake time, shorter non-rapid eye movement sleep (NREMS) time, and longer rapid eye movement sleep (REMS) episodes. After sleep deprivation, males showed an increase in NREMS delta power, NREMS time, and REMS time, but females showed a smaller increase. Females and males showed similar arousal responses. Gonadectomy had only a modest effect on homeostatic sleep regulation in males but enhanced it in females. Gonadectomy weakened arousal response in males and females. With hormone replacement, baseline sleep in gonadectomized females was similar to that of intact females, and baseline sleep in gonadectomized males was close to that of intact males. Gonadal hormone supplementation restored arousal response in males but not in females. These results indicate that male and female mice differ in their baseline sleep–wake behavior, homeostatic sleep regulation, and arousal responses to external stimuli, which are differentially affected by reproductive hormones.

## Introduction

Sleep is regulated in a homeostatic manner so that the drive for sleep becomes stronger after sleep deprivation, resulting in compensatory increases in sleep intensity and sleep amount (Franken et al., 2001; Suzuki A. et al., 2013). Another important feature of sleep control is the flexibility of arousal levels depending on ambient stimuli, which allows animals to increase their odds of escaping predators and acquiring food. The exposure to a novel cage serves as a strong arousal stimuli for mice (Suzuki A. et al., 2013). So far, it is known that male C57BL/6 mice exhibit shorter wake time and longer non-rapid eye movement sleep (NREMS) time than females (Koehl et al., 2006; Paul et al., 2006; Funato et al., 2016). Paul et al. (2006) showed that gonadectomy of male and female mice reduced sex difference in sleep under baseline condition and after sleep deprivation, and Paul et al. (2009a) showed the effects of testosterone or estradiol supplementation on baseline sleep and NREMS delta power after sleep deprivation. However, it remains largely unclear whether there are sex differences in the homeostatic regulation of sleep and the arousal response to external stimuli and whether testosterone or estradiol supplementation can fully restore sleep changes caused by gonadectomy. We have recently reported on sleep/wakefulness during the course of pregnancy in female mice and showed that sleep/wake changes are very gradual until the late stage of pregnancy despite the increase in gonadal hormones caused by pregnancy (Komiya et al., 2018). Although estrogen is generally thought to enhance wakefulness in mice and rats (Paul et al., 2009a; Mong and Cusmano, 2016), whether gonadal hormones are involved in homeostatic sleep regulation and arousal remains largely unknown.

In the present study, we examined sleep/wake behaviors of male and female C57BL/6 mice under the baseline condition using hormonally intact and gonadectomized mice. Intact females showed longer wake time and longer REMS episode duration than males. After gonadectomy, sex differences in sleep were reduced but not eliminated. Intact females were less affected by 6-h sleep deprivation by gentle handling in terms of NREMS delta power and sleep time than intact males. Arousal response to repeated cage changes was reduced after gonadectomy in both males and females. To further examine the effects of gonadal hormones on sleep, castrated males and ovariectomized females were supplemented with testosterone and estradiol, respectively. The results showed that the effect of sex hormones depends on the parameters of sleep.

## Materials and Methods

### Animals

Male and female C57BL/6N mice (CLEA Japan) were used. Mice were housed under a 12-h light/dark cycle with controlled temperature and humidity conditions. Food and water were provided *ad libitum*. All animal experiments in this study were approved (approved protocol ID #21-313) and conducted following the guidelines established by the Institutional Animal Care and Use Committee of the University of Tsukuba.

### Electroencephalogram/Electromyography Electrode Implantation

For intact, gonadectomized, and hormone-supplemented groups, mice were implanted electroencephalogram (EEG)/electromyography (EMG) electrode at 7 weeks of age as described previously (Miyoshi et al., 2019), under anesthesia with isoflurane (4% for induction and 1% for maintenance). Each mouse was implanted with an EEG/EMG electrode containing four electrode pins and two flexible stainless steel wires. EEG/EMG electrodes were lowered to the dura under stereotaxic control. EEG electrodes were positioned over the frontal and occipital cortices [antero-posterior (AP): 0.5 mm, medio-lateral (ML): 1.27 mm, and dorso-ventral (DV): -1.3 mm; AP: -4.5 mm, ML: 1.27 mm, DV: -1.3 mm] and subsequently attached to the skull using dental cement (3M ESPE, RelyX U200 Unicem Syringe Dental Resin Cement). EMG wires were inserted into the neck extensor muscles. Mice were allowed at least 7 days to recover from the surgery. After recovery, mice were individually housed and tethered to counterbalanced arm (Instech), which allows free movement and exerted minimal weight for at least 7 days to habituate to the recording condition.

### Gonadectomy/Hormone Supplementation

For gonadectomized and hormone-supplemented groups, male and female mice were gonadectomized bilaterally during EEG/EMG electrode implantation surgery. Incision was made at the lower abdomen and testes were pulled out and removed. In female mice, dorsal incision above the location of the ovary was made through skin and muscle layers. Ovaries were pulled out to the surface and removed. Incisions were stitched, and the mice were given 3 weeks of recovery period before EEG/EMG recording.

For gonadal hormone supplementation, EEG/EMG-recorded gonadectomized mice (11 weeks old) were implanted with crystalline testosterone-filled (2.0 mg; FUJIFILM Wako Pure Chemical Corporation, Japan) or β-estradiol-filled (0.3 mg; Sigma-Aldrich, Japan) silicone tube (diameter outer 2.0 mm; inner 1.5 mm; Taiyo Kogyo, Japan) (Modder et al., 2004; Suzuki E. et al., 2013; Möller et al., 2016; Müller et al., 2018). Each silastic tube was filled by a funnel made from tip of Pasteur pipette and sealed using a silicone adhesive (TES3941, Momentive Performance Materials Japan, Japan). Mice were anesthetized with isoflurane, and hormone tube was inserted *via* a small dorsal incision made through the skin. Incision was stitched, and the mice were given 3 weeks of recovery period before EEG/EMG sleep recording. The effects of testosterone or estradiol supplementations were confirmed by the restoration of male sexual behavior toward females (Supplementary Figures 1A,B) and by the restoration of uterine weight in ovariectomized females (Supplementary Figure 1C), respectively. The estrous cycle of sham-operated females was not considered. Mice tested for the effect of hormone supplementations were not included in the sleep experiments.

### Uterine Weight

C57BL/6N gonadectomized female mice (8 weeks old) were implanted a silicone tube with different doses of estradiol (0, 0.3, 0.5, or 0.7 mg) according to a previous study (Modder et al., 2004). Sham-operated mice were anesthetized, and dorsal incision was made and stitched without further procedure (Supplementary Figure 1C). At 12 weeks of age, mice were sacrificed with cervical dislocation under isoflurane anesthesia. The uteri were excised and weighed. Ovariectomy resulted in reduced uterine weight, and supplementation of estradiol restored uterine weight to that to the same level as in sham-operated females. Female mice used in the uteri weighing were not included in the sleep experiments.

### Male Sexual Behavior

C57BL/6N gonadectomized male mice (8 weeks old) were implanted a silicone tube with different doses of testosterone (0, 0.5, 2, or 9 mg). At 12 weeks of age, testosterone tube-implanted gonadectomized male mice were assessed for sexual behaviors during exposure to receptive female mice as previously reported (Fujiyama et al., 2018). All tested male mice were housed individually in a home cage, and a female mouse was placed in the cage. The number of male sexual behaviors (mounting attempts and mounting) was counted for 30 min under dim red illumination. Male mice behavior of straddling the female from behind and continuation of hip thrusting was considered as mounting and dismounting before any thrusting movement was considered as mounting attempts.

### Sleep Recording and Data Analysis

Recording environment was kept under a 12-h light/12-h dark cycle and a constant temperature (24–25°C). EEG/EMG signaling was obtained and analyzed as previously described with some modifications (Miyoshi et al., 2019). EEG/EMG signals were amplified, filtered (EEG: 0.3–300 Hz; EMG: 30–300 Hz) with a multichannel amplifier (NIHON KODEN, #AB-611J), digitized at 250-Hz sampling rate using an analog-to-digital converter (National Instruments #PCI-6220) and LabView (National Instruments)-based custom-made software. EEG/EMG data were visualized and semiautomatically analyzed using a MATLAB (MathWorks)-based, custom semi-automated staging program, which allows classification of 20-s epoch time windows into NREMS, REMS, and wakefulness, followed by visual inspection. Wakefulness scoring criteria include high-amplitude and variable EMG. NREMS was scored based on high-amplitude (1–4 Hz) frequency EEG and low EMG tonus, and REMS was characterized by theta (6–9 Hz)-dominant EEG oscillations and EMG atonia. Total time spent in wakefulness, NREMS, and REMS was derived from summing the total number of 20-s epochs in each state. Epochs that contain movement artifacts were included in the state totals but excluded from subsequent spectral analysis. EEG signals were subjected to fast Fourier transform analysis from 1 to 30 Hz with a 1-Hz bin using MATLAB-based custom software. The hourly delta density during NREMS indicates the hourly averages of delta density, which is the ratio of delta power (1–4 Hz) to total EEG power (1–30 Hz) at each 20-s epoch or all epochs. NREMS delta power change was calculated as hourly average of delta power after sleep deprivation divided by that of the same ZT of the baseline recording.

### Recording Schedule

Mice were randomly assigned to gentle handling or cage change groups. EEG/EMG was recorded for three consecutive days. Mean values of the first and second days were used as baseline sleep/wakefulness. Gentle handling or cage change was performed from ZT0 on day 3. Gentle handling was performed during the first 6 h of the light phase (ZT0–6) to deprive of sleep by gentle cage tapping when mice start to recline and lower their head as previously described (Suzuki A. et al., 2013). In the cage change group, the mice were replaced with a new cage once every hour from ZT0 to ZT5 without additional interventions to keep mice awake. During gentle handling or cage change, food and water were available. After gentle handling or cage change, mice were left to sleep freely during the recovery sleep. After sleep recording, gonadectomized mice were subject to gonadal hormone supplementation. Three weeks later, EEG/EMG was recorded for three consecutive days with gentle handling or cage change as described above. EEG/EMG recordings for intact, gonadectomized, and hormone-supplemented mice were conducted at 9, 11, and 15 weeks of age, respectively.

### Statistics

**FIGURE 1:**
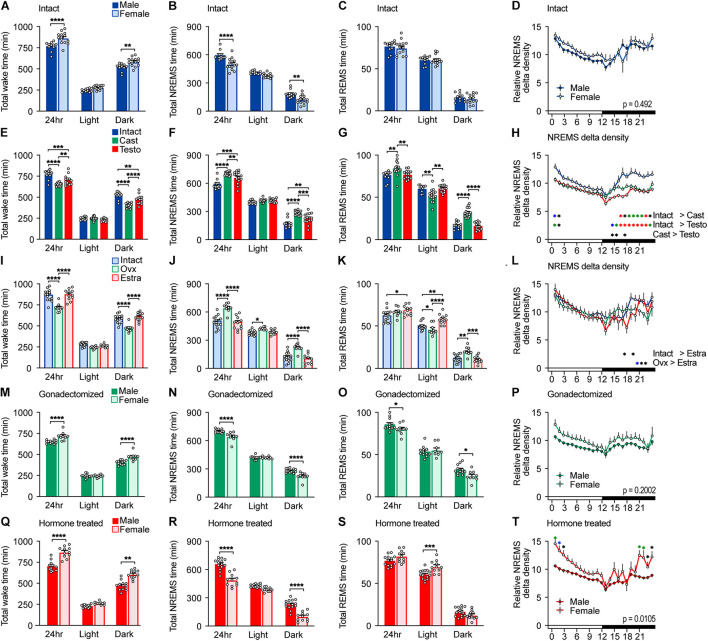
Sleep/wake behavior of intact, gonadectomized, and hormone-supplemented mice. **(A–D)** Total wake time **(A)**, total non-rapid eye movement sleep (NREMS) time **(B)**, total REMS time **(C)**, and NREMS delta power density **(D)** of intact male and female mice for 24 h; 11 male mice and 14 female mice. Two-way ANOVA followed by Sidak’s test. **(E–H)** Total wake time **(E)**, total NREMS time **(F)**, total REMS time **(G)**, and NREMS delta power density **(H)** of male mice. Two-way ANOVA followed by Sidak’s test. **(I–L)** Total wake time **(I)**, total NREMS time **(J)**, total REMS time **(K)**, and NREMS delta power density **(L)** of female mice. Two-way ANOVA followed by Sidak’s test. **(M–P)** Total wake time **(M)**, total NREMS time **(N)**, total REMS time **(O)**, and NREMS delta power density **(P)** of gonadectomized male and female mice; 16 male mice and 14 female mice. Two-way ANOVA followed by Sidak’s test. **(Q–T)** Total wake time **(Q)**, total NREMS time **(R)**, total REMS time **(S)**, and NREMS delta power density **(T)** of gonadal hormone-supplemented gonadectomized male and female mice; 14 male mice and 11 female mice. Two-way ANOVA followed by Sidak’s test. *P < 0.05; **P < 0.01; ****P < 0.0001. **(H,L,T)** *P < 0.05 (black); *P < 0.01 (blue); *P < 0.001 (green); *P < 0.0001 (red).

**FIGURE 2:**
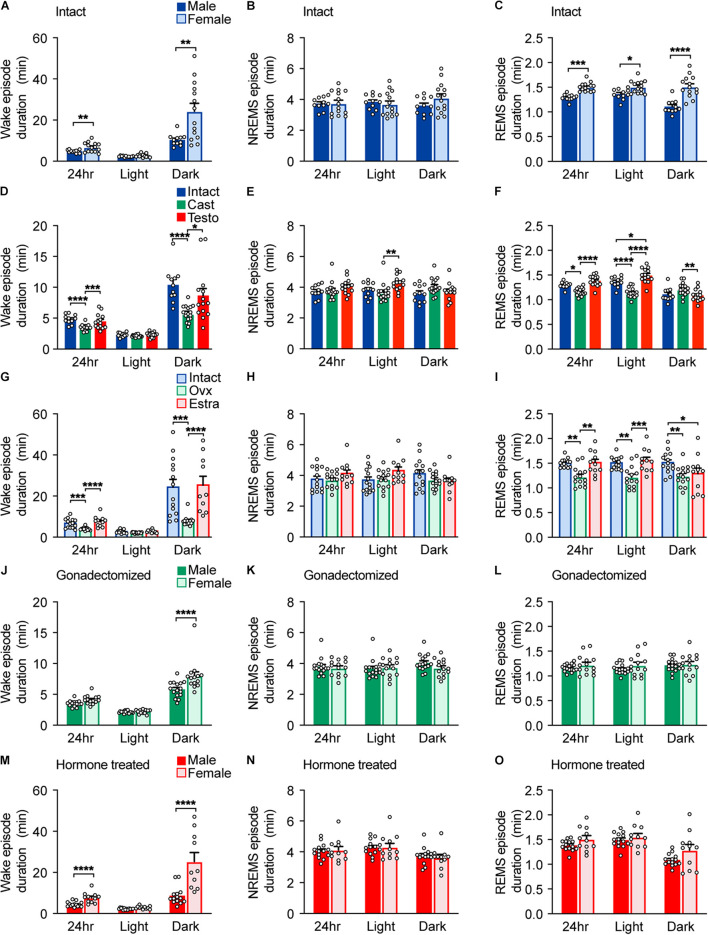
Episode durations of intact, gonadectomized, and hormone-supplemented mice. **(A–C)** Episode durations of wakefulness **(A)**, NREMS **(B)**, and REMS **(C)** of intact male and female mice; 11 male mice and 14 female mice. Two-way ANOVA followed by Sidak’s test or Mann–Whitney *U* test). **(D–F)** Episode durations of wakefulness **(D)**, NREMS **(E)**, and REMS **(F)** of male mice. Two-way ANOVA followed by Sidak’s test or Kruskal–Wallis test. **(G–I)** Episode durations of wakefulness **(G)**, NREMS **(H)**, and REMS **(I)** of female mice. Two-way ANOVA followed by Sidak’s test or Kruskal–Wallis test. **(J–L)** Episode durations of wakefulness **(J)**, NREMS **(K)**, and REMS **(L)** of gonadectomized male and female mice; 16 male mice and 14 female mice. Two-way ANOVA followed by Sidak’s test. **(M–O)** Episode durations of wakefulness **(M)**, NREMS **(N)**, and REMS **(O)** of gonadal hormone-supplemented gonadectomized male and female mice; 14 male mice and 11 female mice. Two-way ANOVA followed by Sidak’s test or Mann–Whitney *U* test. *P < 0.05; **P < 0.01; ***P < 0.001, ****P < 0.0001.

**FIGURE 3:**
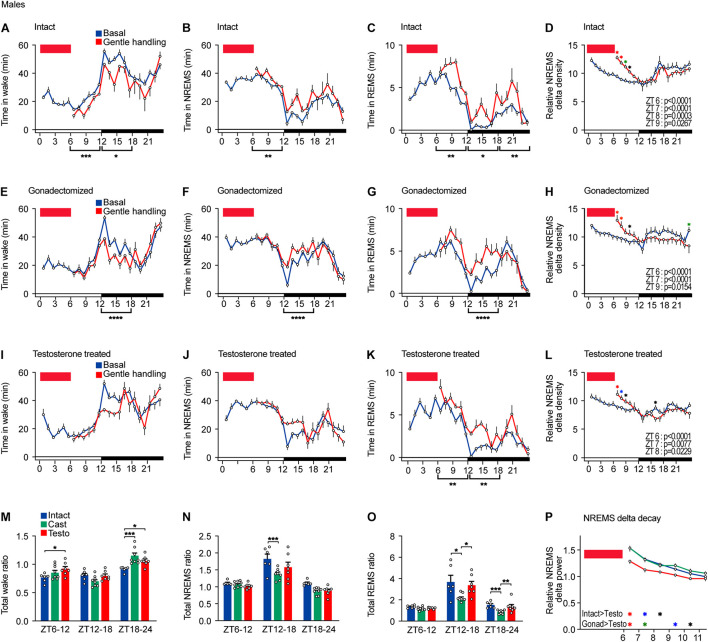
Sleep/wake behavior of male mice after sleep deprivation by gentle handling. **(A–D)** Hourly wake time **(A)**, NREMS time **(B)**, REMS time **(C)**, and NREMS delta density **(D)** under the baseline condition and after 6-h sleep deprivation from ZT0 in intact male mice; six mice. Two-way ANOVA followed by Sidak’s test. **(E–H)** Hourly wake time **(E)**, NREMS time **(F)**, REMS time **(G)**, and NREMS delta density **(H)** under the baseline condition and after 6-h sleep deprivation from ZT0 in gonadectomized male mice; nine mice. Two-way ANOVA followed by Sidak’s test. **(I–L)** Hourly wake time **(I)**, NREMS time **(J)**, REMS time **(K)**, and NREMS delta density **(L)** under the baseline condition and after 6-h sleep deprivation from ZT0 in testosterone-supplemented gonadectomized male mice; seven mice. Two-way ANOVA followed by Sidak’s test. **(M–O)** Ratio of total awake time **(M)**, NREMS time **(N)**, and REMS **(O)** in 18 h to baseline condition after 6 h of sleep deprivation. Two-way ANOVA followed by Sidak’s test or Kruskal–Wallis test. **(P)** NREMS delta after sleep deprivation. Red rectangles in the graphs indicate gentle handling from ZT0 to 6. Two-way ANOVA followed by Sidak’s test. *P < 0.05; **P < 0.01; ***P < 0.001. *P < 0.05 (black); *P < 0.01 (blue); *P < 0.001 (green); *P < 0.0001 (red).

**FIGURE 4:**
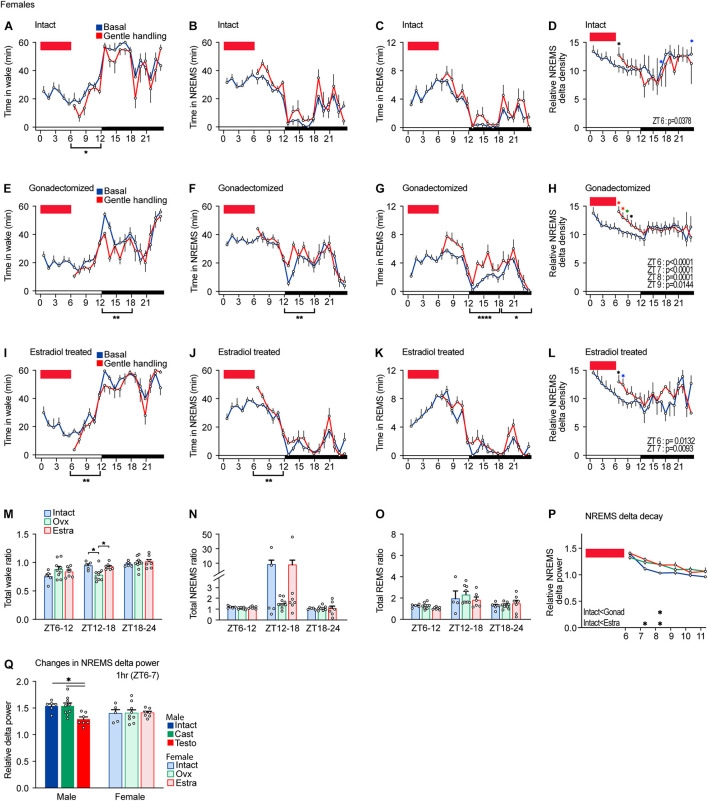
Sleep/wake behavior of female mice after sleep deprivation by gentle handling. **(A–D)** Hourly wake time **(A)**, NREMS time **(B)**, REMS time **(C)**, and NREMS delta density **(D)** under the baseline condition and after 6-h sleep deprivation from ZT0 in intact female mice; five mice. Two-way ANOVA followed by Sidak’s test. **(E–H)** Hourly wake time **(E)**, NREMS time **(F)**, REMS time **(G)**, and NREMS delta density **(H)** under the baseline condition and after 6-h sleep deprivation from ZT0 in gonadectomized female mice; nine mice. Two-way ANOVA followed by Sidak’s test. **(I–L)** Hourly wake time **(I)**, NREMS time **(J)**, REMS time **(K)**, and NREMS delta density **(L)** under the baseline condition and after 6-h sleep deprivation from ZT0 in estradiol-supplemented gonadectomized female mice; seven mice. Two-way ANOVA followed by Sidak’s test. **(M–O)** Ratio of total awake time **(M)**, NREMS time **(N)**, and REMS **(O)** in 18 h to baseline condition after 6 h of sleep deprivation. Two-way ANOVA followed by Sidak’s test or Kruskal–Wallis test. **(P)** NREMS delta after sleep deprivation. Two-way ANOVA followed by Sidak’s test. **(Q)** NREMS delta power for 1 h after 6-h sleep deprivation normalized by the baseline condition. Red rectangles in the graphs indicate gentle handling from ZT0 to 6. Two-way ANOVA followed by Sidak’s test. *P < 0.05; **P < 0.01. *P < 0.05 (black); *P < 0.01 (blue); *P < 0.001 (green); *P < 0.0001 (red).

**FIGURE 5:**
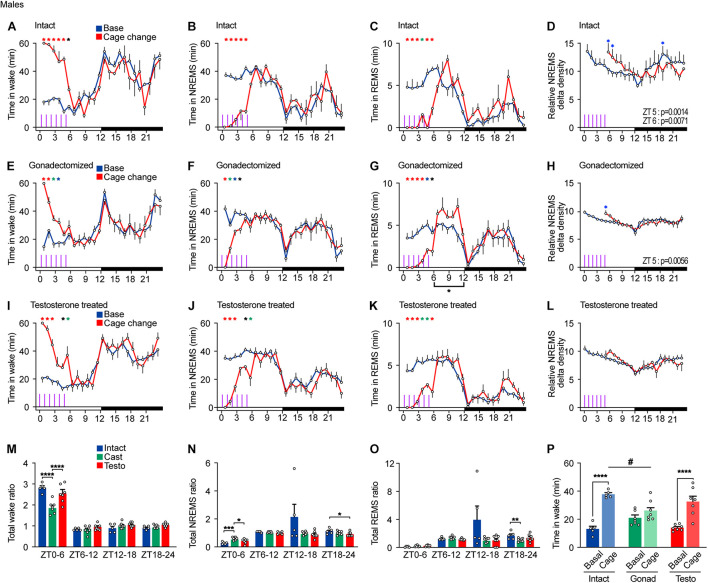
Sleep/wake behavior of male mice in response to repeated cage changes. **(A–D)** Hourly wake time **(A)**, NREMS time **(B)**, REMS time **(C)**, and NREMS delta density **(D)** under the baseline condition and with six hourly cage changes from ZT0 in intact male mice; five mice. Two-way ANOVA followed by Sidak’s test. **(E–H)** Hourly wake time **(E)**, NREMS time **(F)**, REMS time **(G)**, and NREMS delta density **(H)** under the baseline condition and with six hourly cage changes from ZT0 in gonadectomized male mice; seven mice. Two-way ANOVA followed by Sidak’s test. **(I–L)** Hourly wake time **(I)**, NREMS time **(J)**, REMS time **(K)**, and NREMS delta density **(L)** under the baseline condition and with six hourly cage changes from ZT0 in testosterone-supplemented gonadectomized male mice; seven mice. Purple lines indicate cage changes. Two-way ANOVA followed by Sidak’s test. **(M–O)** Ratio of total awake time **(N)**, NREMS time **(O)**, and REMS time **(P)** to the baseline condition after repeated cage changes. Two-way ANOVA followed by Sidak’s test or Kruskal–Wallis test. **(P)** Wake time 2 h from ZT4 under baseline condition and with six hourly cage changes. One-way ANOVA followed by Sidak’s test and two-way ANOVA followed by Sidak’s test. *P < 0.05; **P < 0.01; ***P < 0.001. #*P* < 0.05. *P < 0.05 (black); *P < 0.01 (blue); *P < 0.001 (green); *P < 0.0001 (red).

**FIGURE 6:**
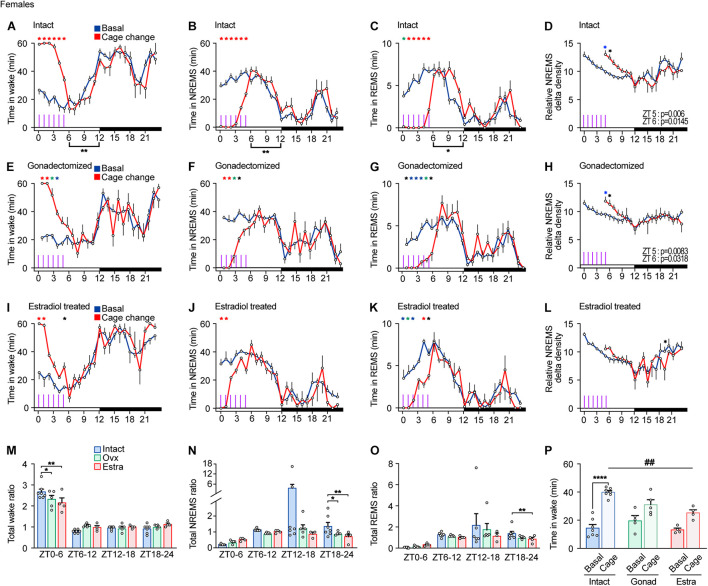
Sleep/wake behavior of female mice in response to repeated cage changes. **(A–D)** Hourly wake time **(A)**, NREMS time **(B)**, REMS time **(C)**, and NREMS delta density **(D)** under the baseline condition and with six hourly cage changes from ZT0 in intact female mice; seven mice. Two-way ANOVA followed by Sidak’s test. **(E–H)** Hourly wake time **(E)**, NREMS time **(F)**, REMS time **(G)**, and NREMS delta density **(H)** under the baseline condition and with six hourly cage changes from ZT0 in gonadectomized female mice; five mice. Two-way ANOVA followed by Sidak’s test. **(I–L)** Hourly wake time **(I)**, NREMS time **(J)**, REMS time **(K)**, and NREMS delta density **(L)** under the baseline condition and with six hourly cage changes from ZT0 in estradiol-supplemented gonadectomized female mice; four mice. Purple lines indicate cage changes. Two-way ANOVA followed by Sidak’s test. **(M–O)** Ratio of total awake time **(M)**, NREMS time **(N)**, and REMS **(O)** to the baseline condition after repeated cage changes. Two-way ANOVA followed by Sidak’s test or Kruskal–Wallis test. **(P)** Wake time for 2 h from ZT4 under baseline condition and with six hourly cage changes. One-way ANOVA followed by Sidak’s test and Two-way ANOVA followed by Sidak’s test. *P < 0.05; **P < 0.01; ***P < 0.001. ##*P* < 0.01. *P < 0.05 (black); *P < 0.01 (blue); *P < 0.001 (green); *P < 0.0001 (red).

Statistical analyses were performed using SPSS Statistics 26 (IBM). All data were tested for Gaussian distribution and variance. Homogeneity of variance was tested with Levene’s test. We used Student’s *t*-test for pairwise comparisons, one-way repeated-measures ANOVA for multiple comparisons with multiple data points, and two-way ANOVA for multiple comparisons involving two independent variables. ANOVA analyses were followed by Sidak’s test. For non-normative data, Mann–Whitney *U* test and Kruskal–Wallis were performed for two-group and three-group comparisons, respectively. Statistical values for Figures 1–6 and Supplementary Figure 2 are summarized in Supplementary Table 1. *P* < 0.05 was considered statistically significant.

## Results

### Effect of Gonadal Hormones on Baseline Sleep/Wakefulness

Male C57BL/6 mice showed shorter total wake time (male, 768 ± 16 min/24 h; female, 865 ± 18 min/24 h) and longer total NREMS time (male, 596 ± 15 min/24 h; female, 500 ± 17 min/24 h) than female mice by approximately 100 min, which was mainly due to differences during the dark phase (Figures 1A,B and Supplementary Table 2). Sleep time of intact females is consistent with our previous report (Komiya et al., 2018). There was no significant difference in total REMS time (male 76.3 ± 1.9 min/24 h; female, 74.7 ± 2.3 min/24 h) (Figure 1C) and NREMS delta density between males and females (Figure 1D). Male C57BL/6 mice showed shorter wake episode duration than females (Figure 2A) as previously reported (Paul et al., 2006) without difference in episode number (Supplementary Figure 2A). Whereas NREMS episode duration and number were similar between males and females (Figure 2B and Supplementary Figure 2B), REMS episode duration of females was longer than that of males with lower episode number (Figure 2C and Supplementary Figure 2C).

To examine the role of gonads in sleep/wakefulness, we performed gonadectomy for males and females. In males, castration resulted in a decrease in daily total wake time and wake episode duration (Figures 1E, 2D), an increase in daily total NREMS time without changes in NREMS episode duration (Figures 1F, 2E), and an increase in daily total REMS time with shorter REMS episode duration (Figures 1G, 2F). Castration increased the number of episodes of wake, NREMS, and REMS in males (Supplementary Figures 2D–F). Castration also led to a decrease in NREMS delta density mainly during the dark phase (Figure 1H). In females, ovariectomy resulted in a decrease in daily total wake time (Figure 1I) with shorter wake episode duration (Figure 2G) and an increase in daily total NREMS time (Figure 1J) without changes in NREMS episode duration (Figure 2H), compared to intact females. Ovariectomized females showed shorter REMS episode duration without changes in daily total REMS time (Figures 1K, 2I). Ovariectomy increased the number of episodes of wake, NREMS, and REMS in females (Supplementary Figures 2G–I). Ovariectomy did not change NREMS delta density (Figure 1L). Thus, the presence of gonads enhances baseline wakefulness in both males and females.

Compared to ovariectomized females, castrated males still showed shorter total wake time (male, 651 ± 5 min/24 h; female, 718 ± 13 min/24 h) and longer total NREMS time (male, 703 ± 4.8 min/24 h; female, 644 ± 12.8 min/24 h) for 24 h and during the dark phase (Figures 1M,N) and shorter wake episode duration during the dark phase without difference in the number of episodes (Figure 2J and Supplementary Figure 2J). Gonadectomized males showed longer total REMS time for 24 h (male, 84.6 ± 2.0 min/24 h; female, 77.3 ± 1.8 min/24 h) and longer REMS time during the dark phase compared to females (Figure 1O), which was due to an increased number of REMS episodes during the dark phase (Supplementary Figure 2L). There was no significant difference in NREMS delta power, NREMS episode duration, NREMS episode number, and REMS episode duration between gonadectomized males and females (Figures 1P, 2K,L and Supplementary Figure 2K). Thus, gonadectomy did not eliminate sex differences in NREMS amount but rather created REMS amount difference during the dark phase while eliminating sex differences in REMS episode duration.

Next, to examine the effect of gonadal hormones, gonadectomized males and females were supplemented with testosterone and estradiol, respectively. The effects of testosterone and estradiol supplementation were confirmed by male sexual behavior and uterine weight, respectively (Supplementary Figure 1). Testosterone supplementation of castrated males led to an increase in total wake time and a decrease in total NREMS and REMS time compared to castrated males (Figures 1E–G). However, total NREMS time of castrated males with testosterone supplementation was still longer than that of intact males (Figure 1F). Similarly, testosterone supplementation did not affect NREMS delta density of castrated males (Figure 1H). Thus, the changes in NREMS time and NREMS delta density in castrated mice were partially restored by testosterone supplementation. In contrast, testosterone supplementation restored REMS changes induced by castration (Figure 1G). Estradiol supplementation of ovariectomized females resulted in an increase in daily wake time (Figure 1I) with longer wake episode duration (Figure 2G) and a decrease in daily NREMS time (Figure 1J) without changes in NREMS episode duration (Figure 2H), compared to ovariectomized mice. Estradiol-supplemented ovariectomized females showed a decrease in REMS time and REMS episode number during the dark phase with increased daily REMS episode duration, compared to ovariectomized females (Figures 1K, 2I and Supplementary Figure 2I). Different from males, estradiol supplementation of ovariectomized females exhibited sleep/wakefulness similar to intact females, except for REMS time especially during the light phase (Figures 1I–L). Thus, estradiol supplementation almost completely reversed the effect of ovariectomy.

Testosterone-supplemented male mice showed shorter total wake time and longer total NREMS time for 24 h, and during the dark phase, a shorter wake episode duration and a lower number of wake and NREMS episodes than estradiol-supplemented female mice (Figures 1Q,R, 2M and Supplementary Figure 2M,N). There was no significance in NREMS episode duration (Figure 2N). Hormone-supplemented mice exhibited similar total REMS time, REMS episode duration, and REMS episode number for 24 h, whereas testosterone-supplemented males showed shorter REMS time during the light phase compared to estradiol-supplemented females (Figures 1S, 2O and Supplementary Figure 2O). Hormone supplementation eliminated the difference in REMS amount in the dark phase, especially the first half of the dark phase, which was induced by gonadectomy (Figure 1S and Supplementary Table 2). Testosterone-supplemented male mice showed lower NREMS delta density than estradiol-supplemented female mice (Figure 1T). For intact mice, there was no significant difference in coefficient of variation of total time spent in wake (male, 0.0498 ± 0.0137; female, 0.0623 ± 0.0123; *p* = .504), NREMS (male 0.602 ± 0.0164; female, 0.0961 ± 0.0207; *p* = .205), and REMS (male, 0.0679 ± 0.0147; female, 0.096 ± 0.0176; *p* = 0.25). Thus, male mice showed longer NREMS time than female mice in three different gonadal hormonal conditions, but the largest difference was found in hormone-supplemented gonadectomized groups (difference in mean: 95.37 min for intact, 59.19 min for gonadectomized, and 169.73 min for hormone supplemented).

### Sleep/Wake Behavior of Male Mice After Sleep Deprivation by Gentle Handling

To examine homeostatic regulation of sleep of mice with different hormonal conditions, we performed sleep deprivation for 6 h from ZT0 by gentle handling. After sleep deprivation, intact males showed a slight decrease in wake time and an increase in NREMS and REMS time during the remaining light phase (ZT6–12), but NREMS ratio normalized by baseline NREMS revealed the increase in NREMS was more prominent during the early dark phase (Figures 3A,B,M,N). After sleep deprivation, total REMS time increased during the remaining light phase and the dark phase (Figures 3C,O). Sleep deprivation led to an immediate increase in NREMS delta power, which was followed by a gradual decrease to the basal level, and no difference during the dark phase (Figure 3D). Thus, sleep deprivation results in an immediate increase in NREMS delta power and NREMS time and a prolonged increase in REMS time throughout the dark phase.

After sleep deprivation, castrated males showed shorter wake time, longer NREMS time, and longer REMS time during the early dark phase compared to the basal condition (Figures 3E–G). However, the increased ratios of NREMS time and REMS time during the early dark phase (ZT12–18) were lower than those of intact males (Figures 3N,O). Gonadectomized males showed an increase in NREMS delta power after sleep deprivation similar to intact males (Figures 3H,P). In testosterone-supplemented castrated mice, sleep deprivation did not lead to any change in total wake time and NREMS time during the remaining light phase compared to the basal condition (Figures 3I,J,M,N). Testosterone-supplemented mice showed an increase in NREMS time and REMS time during the early dark phase (ZT12–18) similar to intact males (Figures 3J,K,N,O). The increase in NREMS delta power of testosterone-supplemented castrated males was weaker than that of intact males and of castrated males (Figures 3L,P). Thus, compared to intact males, castration weakened the increase in NREMS time and REMS time, but not NREMS delta power, in response to sleep deprivation in males. Testosterone supplementation normalized NREMS time increase and REMS time increase to the level of intact males.

### Sleep/Wake Behavior of Female Mice After Sleep Deprivation by Gentle Handling

Intact females showed a decrease in total wake time during the light phase after sleep deprivation (Figure 4A). However, intact females did not show significant changes in time spent in NREMS and REMS during the light phase and all wake, NREMS, and REMS during the dark phase in response to sleep deprivation (Figures 4A–C). Sleep deprivation for 6 h led to an increase in NREMS delta only at ZT6 (Figure 4D). Thus, the effect of sleep deprivation on females was much weaker than males for both NREMS and REMS.

After sleep deprivation, gonadectomized females showed a decrease in wake time and an increase in NREMS time and REMS time during the dark phase compared to the basal condition (Figures 4E–G). Sleep deprivation led to an increase in NREMS delta from ZT6 to 9 (Figure 4H). Thus, gonadectomized females exhibited a stronger response to sleep deprivation compared to intact females in terms of sleep time and NREMS delta power. Estradiol-supplemented females showed a decrease in wake time and an increase in NREMS mainly during the dark phase (Figures 4I,J). The increase in REMS time was not significant (Figure 4K). The increase in NREMS delta power of estradiol-supplemented gonadectomized females was longer (ZT6 and 7) than that of intact males (ZT6) but shorter than that of gonadectomized males (ZT6–9) (Figure 4L). However, the time ratio normalized by the baseline sleep/wakefulness did not show significant changes except for wake time during the early dark phase (Figures 4M–O). One female in the intact group and one female in the estradiol-supplemented group showed very high total NREMS ratios at ZT12–18, but the difference was not significant (Figure 4N). NREMS delta decay of intact females was faster than that of ovariectomized and estradiol-supplemented females (Figure 4P). The extent of NREMS delta increase right after sleep deprivation in females was similar among three hormonal conditions and was similar to males (Figure 4Q). Thus, compared to intact females, ovariectomy enhanced the response to sleep deprivation in recovery sleep time, and NREMS delta power and estradiol supplementation partially reversed the effects of ovariectomy.

### Sleep/Wake Behavior of Male Mice in Response to Repeated Cage Changes

Next, we examined arousal response to repeated cage changes every hour from ZT0 to ZT5 in males. Intact males exhibited a strong arousal response to the first five cage changes and a weaker response in wake to the sixth cage changes (Figures 5A,B). The first to sixth cage changes strongly suppressed REMS in intact males (Figure 5C). Repeated cage changes led to an increase in NREMS delta density at ZT5 and 6 (Figure 5D).

Castrated males showed a strong arousal response to the first and second cage changes but a weaker response to the third change and thereafter. Castrated males did not show arousal response to the fifth and sixth cage changes (Figures 5E,F). In contrast, REMS of gonadectomized males was suppressed by repeated cage changes. Even the sixth cage change moderately suppressed REMS, which was followed by a compensatory increase in REMS time during the late light phase (Figure 5G). Repeated cage changes led to an increase in NREMS delta density at ZT5 (Figure 5H). Testosterone-supplemented castrated males showed a moderate arousal response to the fifth and sixth cage changes (Figures 5I,J). Repeated cage changes suppressed REMS in testosterone-supplemented castrated males, which was followed by a slight compensatory increase in REMS time during the late light phase (Figure 5K). In testosterone-supplemented males, repeated cage changes did not lead to an increase in NREMS delta density (Figure 5L). Among the three hormonal conditions, castrated males showed a smaller wake time increase ratio and a greater NREMS time increase ratio in response to repeated cage changes compared to intact males and testosterone-supplemented males (Figures 5M,N). Wake time for 2 h from ZT4 during the cage changes of castrated males was shorter than that of intact males (Figure 5P). During and immediately after repeated cage changes (ZT0–6), REMS in male mice was strongly suppressed under all hormonal conditions (Figure 5O). Thus, castration reduced arousal response in cage change-induced wake amount and NREMS delta density, and testosterone supplementation partially reversed the effects of castration except for NREMS delta density and stability of response to repeated cage changes.

### Sleep/Wake Behavior of Female Mice in Response to Repeated Cage Changes

Lastly, we examined arousal response to repeated cage changes every hour from ZT0 to ZT5 in females. Intact females exhibited a strong wake response to all cage changes, showing moderate response even to the last cage changes (Figures 6A,B,M,N). REMS in intact female was strongly suppressed in all six cage changes (Figures 6C,O). Repeated cage changes increased NREMS delta power at ZT5 and 6 (Figure 6D).

Gonadectomized females exhibited a strong arousal response to the first to third cage changes but a weaker response to the fourth change (Figures 6E,F). REMS in gonadectomized females was continuously suppressed by repeated cage changes (Figure 6G). Repeated cage changes increased NREMS delta power at ZT5 and 6 (Figure 6H). Estradiol-supplemented gonadectomized females showed a strong arousal response to the first and second cage changes and a weaker response thereafter (Figures 6I,J). Repeated cage changes suppressed REMS throughout the period in estradiol-supplemented ovariectomized females (Figure 6K). Estradiol-supplemented gonadectomized females did not show an increase in NREMS delta power after the cage changes (Figure 6L). After repeated cage changes, the total NREMS time increase during the dark phase was higher in intact female compared to other groups (Figure 6N). During cage changing, repeated cage changes strongly suppressed REMS in female mice under all hormonal conditions (Figure 6O). Wake time for 2 h from ZT4 during the cage changes of estradiol-supplemented females was not significantly higher than the basal amount and was shorter than that of intact females (Figure 6P). There is no significant difference in wake time for 2 h from ZT4 during the cage changes between intact males and females (Student’s *t*-test, *P* = 0.935), castrated males and ovariectomized females (*P* = 0.549), and hormone-supplemented males and females (*P* = 0.247). Thus, compared to intact females, ovariectomy weakened arousal response in cage change-induced wake amount and wake and NREMS response stability, and estradiol supplementation did not reverse the effects of ovariectomy.

## Discussion

As an animal model, inbred mice have been used because they can produce reproducible results and are suitable for genetic manipulation. Among various inbred strains of mice, the C57BL/6 line strain has been used as a common platform of mutant mice for various physiological and behavioral studies (Beck et al., 2000; Skarnes et al., 2011; Tam and Cheung, 2020). In sleep research, C57BL/6 mice have contributed to our understanding of sleep regulation at the circuit and molecular levels (Cirelli, 2009; Liu and Dan, 2019). However, most of the studies have been conducted using male C57BL/6 mice, not female mice, and this male-biased research results may hinder the characterization of sexually dimorphic effects (Cahill, 2006), which may lead to an underestimation of risks for women, such as adverse reaction to drugs (Clayton and Collins, 2014), and it remains unclear whether the findings are specific to males or valid for both sexes.

The present study shows sex differences in baseline sleep, the homeostatic regulation of sleep, and arousal response to a novel cage in C57BL/6 mice. For baseline sleep, female mice spent more time in wakefulness, less time in NREMS, and a similar amount of time in REMS compared to male mice as previously reported (Paul et al., 2006; Ehlen et al., 2013; Funato et al., 2016). As NREMS delta density was similar between males and females, the shorter NREMS time in female by 100 min is not compensated for by higher delta power during NREMS. Thus, female mice have a lower need in terms of sleep amount under the baseline condition. Although there has been no report on sex difference in sleep episode duration and episode number, we showed that female mice have longer REMS episode duration with lower episode number and similar NREMS episode duration and episode number compared to male mice. The mechanism to determine REMS episode duration remains unknown, but mice with a mutation in sodium leak channel, NALCN, exhibit short REMS episode duration (Funato et al., 2016), suggesting the possible link between gonadal hormone and ion channel in the regulation of REMS.

Sleep deprivation by gentle handling in males increases NREMS delta power and REMS time immediately after the deprivation and NREMS time mainly during the dark phase. In contrast to males, females showed a weak and brief increase in NREMS delta power, with no immediate increase in REMS time or no increase in NREMS time during the dark phase. Importantly, deprived sleep amount during 6-h gentle handling was similar between males and females because NREMS amount during the light phase under the baseline condition is similar between males and females. These findings indicate that females have weaker homeostatic regulation of both NREMS and REMS compared to males. In contrast to weaker responses to sleep deprivation, female mice exhibited an arousal response to repeated cage changes, similar to male mice. These findings indicate arousal and homeostatic regulation of sleep need can be dissociable as we have previously shown (Suzuki A. et al., 2013). *Sik3* mutant mice also showed an increased sleep need despite an intact arousal response to stimuli (Funato et al., 2016).

In males, castration alters sleep/wakefulness under the baseline condition and the responses to sleep perturbation. For baseline sleep/wakefulness, castration increased NREMS time and decreased NREMS delta power. As for the response to sleep deprivation, castration weakened NREMS time increase and REMS time increase during the early dark phase. Regarding repeated cage changes, castrated males exhibited a weaker and shorter wake response. Thus, castrated males have weaker regulation of sleep homeostasis and weaker wake-promoting response to external stimuli, compared to intact males. When castrated males were supplemented with testosterone, REMS time was reduced to the same extent as in intact males, and NREMS time was also reduced, but still longer than intact males. For the response to sleep perturbation, testosterone supplementation normalized NREMS and REMS time increase during the early dark phase after sleep deprivation and arousal response to repeated cage changes. Thus, the effect of castration on most of sleep parameters, especially REMS parameters and response to sleep deprivation and external stimuli, can be explained by the effect of testosterone. In other words, testosterone is involved in baseline NREMS and REMS quantity, NREMS quality, sleep homeostasis, and arousal. However, several sleep parameters such as total wake time during the light phase and NREMS delta decay after sleep deprivation cannot be explained by testosterone in males.

In females, ovariectomy alters sleep/wakefulness under the baseline condition and the responses to sleep perturbation. For baseline sleep/wakefulness, ovariectomy decreased wake time and increased NREMS time as previously reported (Paul et al., 2006). Supplementation with estradiol increased daily wake time and decreased daily NREMS time and REMS time during the dark phase, indicating that the effect of ovariectomy on baseline sleep parameters is primarily due to the lack of estradiol. Aromatase-deficient female mice that are deficient in estrogen showed a decrease in wake time during the dark phase (Vyazovskiy et al., 2006). In addition, gonadectomy increased REMS time during the dark phase and decreased it during the light phase, and these changes were restored by gonadal hormone supplementation, suggesting that gonadal hormone may act to suppress REMS only during the dark phase. The time-of-day effect of gonadal hormones on REMS is consistent with the finding that estradiol suppresses REMS only during the dark phase in rats (Schwartz and Mong, 2013). For the response to sleep deprivation, ovariectomy enhanced NREMS delta increase immediately after sleep deprivation, wake time decrease, and REMS time increase during the dark phase. For repeated cage changes, ovariectomized females exhibited weaker and shorter arousal. Thus, ovariectomized females have reduced NREMS amount, enhanced regulation of sleep homeostasis, and weaker wake-promoting response to external stimuli. Estradiol supplementation to ovariectomized females normalized wake and NREMS time under the baseline condition as previously reported (Paul et al., 2009a). When ovariectomized females were supplemented with estradiol, their response to sleep deprivation was as weak as that of intact mice, revealing an inhibitory role for estradiol in the homeostatic control of sleep. Estradiol supplementation in ovariectomized female rats has also reported to decrease NREMS delta increase from 6-h sleep deprivation (Deurveilher et al., 2009). Estradiol supplementation did not normalize arousal responses to repeated cage changes in ovariectomized females, which suggests that estradiol does not play an important role in wake response to external stimuli.

Current findings clearly show that there are large sex differences in sleep/wakefulness in mice. Since the rodent brain has sexual dimorphism, which underlies sex differences in innate behaviors such as sexual behavior, aggression, and parental behavior (Bonthuis et al., 2010; McCarthy et al., 2017; Tsuneoka et al., 2017a; Fujiyama et al., 2018; Tsuneoka and Funato, 2021), it is possible that sleep/wake behaviors are also controlled by neural groups with sexual dimorphism. Although the current study shows the role of gonadal hormone in sleep/wake regulation, gonadectomy does not eliminate all sex differences in sleep. In agreement with our result, Paul et al. (2006, 2009a) showed the eliminated sex differences in some of the responses to sleep deprivation in gonadectomized mice; recovery sleep amount, for example, can be restored depending on the timing of the sleep perturbation, suggesting sex differences are not fully dependent on gonadal hormones. Similarly in rats, gonadal hormone supplementation in gonadectomized male and female induces clear sex difference in sensitivity to hormonal effect on sleep (Cusmano et al., 2014). Ovariectomized females showed a greater wake amount and a smaller NREMS amount compared to castrated males as previously reported (Ehlen et al., 2013), which can be explained by sex differences in the brain determined before puberty. Exposure to testosterone at the perinatal and early postnatal period or “critical period” in male pups is required for the proper development of sexual dimorphic structures in brains (Bonthuis et al., 2010; McCarthy et al., 2017; Fujiyama et al., 2018). In fact, we previously reported that there is no sex difference in total time spent in wake, NREMS, and REMS in hypothalamic *Ptf1a*-deficient mice, in which sexual dimorphism in the brain is not properly developed (Fujiyama et al., 2018). In the hypothalamus, the medial preoptic area (MPOA) is abundant in estrogen receptors (Tsuneoka et al., 2017b). Androgen receptors are also expressed in the hypothalamus, including the MPOA, and are more abundant in males and less abundant in females (Brock et al., 2015). In male, testosterone also act on estrogen receptors, as well as androgen receptors, through aromatization to estradiol (Purves-Tyson et al., 2012). Given that the MPOA expresses receptors for reproductive hormones and contains neurons that regulate sleep/wakefulness and exhibit sexual dimorphism (Liu and Dan, 2019; Tsuneoka and Funato, 2021), the MPOA may be involved in sex difference in sleep/wakefulness.

In this study, we used estrogen to supplement ovariectomy because estrogen increased wakefulness in ovariectomized mice (Paul et al., 2009a), and estrogen alone or in combination with progesterone increased wakefulness in ovariectomized rats, but progesterone alone did not (Deurveilher et al., 2009), suggesting that estrogen is a major female gonadal hormone that influences sleep/wakefulness. However, progesterone might be involved in the sleep changes exhibited by estradiol-supplemented ovariectomized mice, such as weaker responses to repeated cage changes. Sex chromosome may also play a role in sleep regulation that were not fully restored by testosterone or estrogen supplementation (Ehlen et al., 2013). In addition, sleep deprivation inevitably increases corticosterone level in C57BL/6 mice even when using gentle handling (Mongrain et al., 2010; Suzuki A. et al., 2013). The increase in EEG delta power after sleep deprivation was similar in control mice and adrenalectomized male mice (Mongrain et al., 2010), suggesting that changes in corticosterone levels do not strongly affect sleep. However, it is possible that sex difference in stress response may be involved in sex differences in response to sleep deprivation or cage changes.

Estrous cycle of females was not taken into account as in previous studies (Paul et al., 2006, 2009b). This is because the NREMS and REMS time of C57BL6 mice do not vary with the estrous cycle (Koehl et al., 2003). The variability of total sleep/wake time in the intact mice did not show any sex difference in our study. However, it is still possible that female mice with altered hormone profiles from different stages of estrous cycle have influence on the indices of homeostatic and voluntary response in this study. In summary, male and female mice exhibited distinct baseline sleep/wake behavior, homeostatic sleep regulation, and arousal response to external stimuli, suggesting that sleep studies using female animal models are necessary to understand the universal mechanism of sleep regardless of sex.

## Data Availability Statement

The raw data supporting the conclusions of this article will be made available by the authors, without undue reservation.

## Ethics Statement

The animal study was reviewed and approved by the Institutional Animal Care and Use Committee of the University of Tsukuba.

## Author Contributions

MY and HF conceptualized and supervised the study. JC, SK, TF, MP, HS-A, and CM made significant contributions in the methodology. JC, SK, and HF performed the formal analysis. JC, MY, and HF wrote the manuscript. All authors contributed to the article and approved the submitted version.

## Conflict of Interest

The authors declare that the research was conducted in the absence of any commercial or financial relationships that could be construed as a potential conflict of interest.

## Publisher’s Note

All claims expressed in this article are solely those of the authors and do not necessarily represent those of their affiliated organizations, or those of the publisher, the editors and the reviewers. Any product that may be evaluated in this article, or claim that may be made by its manufacturer, is not guaranteed or endorsed by the publisher.

## References

[B1] Beck J. A. Lloyd S. Hafezparast M. Lennon-Pierce M. Eppig J. T. Festing M. F. (2000). Genealogies of mouse inbred strains. *Nat. Genet.* 24 23–25. 10.1038/71641 10615122

[B2] Bonthuis P. J. Cox K. H. Searcy B. T. Kumar P. Tobet S. Rissman E. F. (2010). Of mice and rats: key species variations in the sexual differentiation of brain and behavior. *Front. Neuroendocrinol.* 31:341–358. 10.1016/j.yfrne.2010.05.001 20457175 PMC2910167

[B3] Brock O. De Mees C. Bakker J. (2015). Hypothalamic expression of oestrogen receptor α and androgen receptor is sex-, age- and region-dependent in mice. *J. Neuroendocrinol.* 27 264–276. 10.1111/jne.12258 25599767

[B4] Cahill L. (2006). Why sex matters for neuroscience. *Nat. Rev. Neurosci.* 7 477–484. 10.1038/nrn1909 16688123

[B5] Cirelli C. (2009). The genetic and molecular regulation of sleep: from fruit flies to humans. *Nat. Rev. Neurosci.* 10 549–560. 10.1038/nrn2683 19617891 PMC2767184

[B6] Clayton J. A. Collins F. S. (2014). Policy: NIH to balance sex in cell and animal studies. *Nature* 509 282–283. 10.1038/509282a 24834516 PMC5101948

[B7] Cusmano D. M. Hadjimarkou M. M. Mong J. A. (2014). Gonadal steroid modulation of sleep and wakefulness in male and female rats is sexually differentiated and neonatally organized by steroid exposure. *Endocrinology* 155 204–214. 10.1210/en.2013-1624 24189140 PMC3868804

[B8] Deurveilher S. Rusak B. Semba K. (2009). Estradiol and progesterone modulate spontaneous sleep patterns and recovery from sleep deprivation in ovariectomized rats. *Sleep* 32 865–877. 19639749 PMC2704917

[B9] Ehlen J. C. Hesse S. Pinckney L. Paul K. N. (2013). Sex chromosomes regulate nighttime sleep propensity during recovery from sleep loss in mice. *PLoS One* 8:e62205. 10.1371/journal.pone.0062205 23658713 PMC3641056

[B10] Franken P. Chollet D. Tafti M. (2001). The homeostatic regulation of sleep need is under genetic control. *J. Neurosci.* 21 2610–2621. 10.1523/jneurosci.21-08-02610.2001 11306614 PMC6762509

[B11] Fujiyama T. Miyashita S. Tsuneoka Y. Kanemaru K. Kakizaki M. Kanno S. (2018). Forebrain Ptf1a is required for sexual differentiation of the brain. *Cell Rep.* 24 79–94. 10.1016/j.celrep.2018.06.010 29972793

[B12] Funato H. Miyoshi C. Fujiyama T. Kanda T. Sato M. Wang Z. (2016). Forward-genetics analysis of sleep in randomly mutagenized mice. *Nature* 539 378–383. 10.1038/nature20142 27806374 PMC6076225

[B13] Koehl M. Battle S. Meerlo P. (2006). Sex differences in sleep: the response to sleep deprivation and restraint stress in mice. *Sleep* 29 1224–1231. 10.1093/sleep/29.9.1224 17040010

[B14] Koehl M. Battle S. E. Turek F. W. (2003). Sleep in female mice: a strain comparison across the estrous cycle. *Sleep* 26 267–272. 10.1093/sleep/26.3.267 12749544

[B15] Komiya H. Miyoshi C. Iwasaki K. Hotta-Hirashima N. Ikkyu A. Kanno S. (2018). Sleep/Wake behaviors in mice during pregnancy and pregnancy-associated hypertensive mice. *Sleep* 41:zsx209. 10.1093/sleep/zsx209 29309677

[B16] Liu D. Dan Y. (2019). A motor theory of sleep-wake control: arousal-action circuit. *Annu. Rev. Neurosci.* 42 27–46. 10.1146/annurev-neuro-080317-061813 30699051

[B17] McCarthy M. M. Nugent B. M. Lenz K. M. (2017). Neuroimmunology and neuroepigenetics in the establishment of sex differences in the brain. *Nat. Rev. Neurosci.* 18 471–484. 10.1038/nrn.2017.61 28638119 PMC5771241

[B18] Miyoshi C. Kim S. J. Ezaki T. Ikkyu A. Hotta-Hirashima N. Kanno S. (2019). Methodology and theoretical basis of forward genetic screening for sleep/wakefulness in mice. *Proc. Natl. Acad. Sci. U.S.A.* 116 16062–16067. 10.1073/pnas.1906774116 31337678 PMC6689935

[B19] Modder U. I. L. Riggs B. L. Spelsberg T. C. Fraser D. G. Atkinson E. J. Arnold R. (2004). Dose-response of estrogen on bone versus the uterus in ovariectomized mice. *Eur. J. Endocrinol.* 151 503–510. 10.1530/eje.0.1510503 15476452

[B20] Möller F. J. Pemp D. Soukup S. T. Wende K. Zhang X. Zierau O. (2016). Soy isoflavone exposure through all life stages accelerates 17β-estradiol-induced mammary tumor onset and growth, yet reduces tumor burden, in ACI rats. *Arch. Toxicol.* 90 1907–1916. 10.1007/s00204-016-1674-2 26861028

[B21] Mong J. A. Cusmano D. M. (2016). Sex differences in sleep: impact of biological sex and sex steroids. *Philos. Trans. R. Soc. Lond. B Biol. Sci.* 371:20150110. 10.1098/rstb.2015.0110 26833831 PMC4785896

[B22] Mongrain V. Hernandez S. A. Pradervand S. Dorsaz S. Curie T. Hagiwara G. (2010). Separating the contribution of glucocorticoids and wakefulness to the molecular and electrophysiological correlates of sleep homeostasis. *Sleep* 33 1147–1157. 10.1093/sleep/33.9.1147 20857860 PMC2938796

[B23] Müller S. T. Keiler A. M. Kräker K. Zierau O. Bernhardt R. (2018). Influence of estrogen on individual exercise motivation and bone protection in ovariectomized rats. *Lab. Anim.* 52 479–489. 10.1177/0023677218756455 29426272

[B24] Paul K. N. Dugovic C. Turek F. W. Laposky A. D. (2006). Diurnal sex differences in the sleep-wake cycle of mice are dependent on gonadal function. *Sleep* 29 1211–1223. 10.1093/sleep/29.9.1211 17040009

[B25] Paul K. N. Laposky A. D. Turek F. W. (2009a). Reproductive hormone replacement alters sleep in mice. *Neurosci. Lett.* 463 239–243. 10.1016/j.neulet.2009.07.081 19647784 PMC2749224

[B26] Paul K. N. Losee-Olson S. Pinckney L. Turek F. W. (2009b). The ability of stress to alter sleep in mice is sensitive to reproductive hormones. *Brain Res.* 1305 74–85. 10.1016/j.brainres.2009.09.055 19769952 PMC2787829

[B27] Purves-Tyson T. D. Handelsman D. J. Double K. L. Owens S. J. Bustamante S. Weickert C. S. (2012). Testosterone regulation of sex steroid-related mRNAs and dopamine-related mRNAs in adolescent male rat substantia nigra. *BMC Neurosci.* 13:95. 10.1186/1471-2202-13-95 22867132 PMC3467168

[B28] Schwartz M. D. Mong J. A. (2013). Estradiol modulates recovery of REM sleep in a time-of-day-dependent manner. *Am. J. Physiol. Regul. Integr. Comp. Physiol.* 305 271–280. 10.1152/ajpregu.00474.2012 23678032 PMC3743004

[B29] Skarnes W. C. Rosen B. West A. P. Koutsourakis M. Bushell W. Iyer V. (2011). A conditional knockout resource for the genome-wide study of mouse gene function. *Nature* 474 337–342. 10.1038/nature10163 21677750 PMC3572410

[B30] Suzuki A. Sinton C. M. Greene R. W. Yanagisawa M. (2013). Behavioral and biochemical dissociation of arousal and homeostatic sleep need influenced by prior wakeful experience in mice. *Proc. Natl. Acad. Sci. U.S.A.* 110 10288–10293. 10.1073/pnas.1308295110 23716651 PMC3690840

[B31] Suzuki E. Eda-Fujiwara H. Satoh R. Saito R. Miyamoto T. (2013). The effect of androgen on the retention of extinction memory after conditioned taste aversion in mice. *J. Physiol. Sci.* 63 171–181. 10.1007/s12576-013-0258-7 23539343 PMC10717145

[B32] Tam W. Y. Cheung K.-K. (2020). Phenotypic characteristics of commonly used inbred mouse strains. *J. Mol. Med.* 98 1215–1234. 10.1007/s00109-020-01953-4 32712726

[B33] Tsuneoka Y. Funato H. (2021). Cellular Composition of the Preoptic Area Regulating Sleep, Parental, and Sexual Behavior. *Front. Neurosci.* 15:327. 10.3389/fnins.2021.649159 33867927 PMC8044373

[B34] Tsuneoka Y. Tsukahara S. Yoshida S. Takase K. Oda S. Kuroda M. (2017a). Moxd1 Is a Marker for Sexual Dimorphism in the Medial Preoptic Area, Bed Nucleus of the Stria Terminalis and Medial Amygdala. *Front. Neuroanat.* 11:26. 10.3389/fnana.2017.00026 28396628 PMC5366752

[B35] Tsuneoka Y. Yoshida S. Takase K. Oda S. Kuroda M. Funato H. (2017b). Neurotransmitters and neuropeptides in gonadal steroid receptor-expressing cells in medial preoptic area subregions of the male mouse. *Sci. Rep.* 7:9809. 10.1038/s41598-017-10213-4 28852050 PMC5575033

[B36] Vyazovskiy V. V. Kopp C. Wigger E. Jones M. E. E. Simpson E. R. Tobler I. (2006). Sleep and rest regulation in young and old oestrogen-deficient female mice. *J. Neuroendocrinol.* 18 567–576. 10.1111/j.1365-2826.2006.01452.x 16867177

