# Supplementary material for: The Role of Reproductive Hormones in Sex Differences in Sleep Homeostasis and Arousal Response in Mice
Source: Front Neurosci. 2021 Sep 21;15:739236. doi: 10.3389/fnins.2021.739236 (PMC8491770; doi:10.3389/fnins.2021.739236)
Supplement: Supplementary file 1 [file Data_Sheet_1.PDF]

Supplementary Material

1 Supplementary Figures

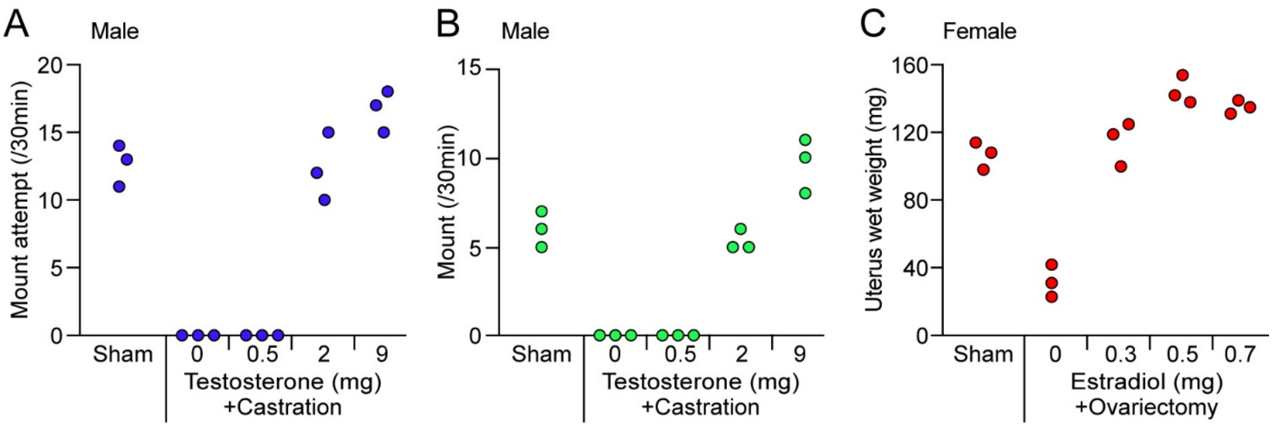

Supplementary Figure 1. (A,B) Male sexual behaviors were scored in castrated males with different amount of testosterone supplementation. Mount attempt (A) and Mount (B) for 30min. 3 mice per group. (C) Female uteri were weighed in ovariectomized females with different amount of estradiol supplementation. 3 mice per group.
