# Supplementary material for: The Role of Reproductive Hormones in Sex Differences in Sleep Homeostasis and Arousal Response in Mice
Source: Front Neurosci. 2021 Sep 21;15:739236. doi: 10.3389/fnins.2021.739236 (PMC8491770; doi:10.3389/fnins.2021.739236)
Supplement: Supplementary file 2 [file Data_Sheet_2.PDF]

Supplementary Material

1 Supplementary Figures

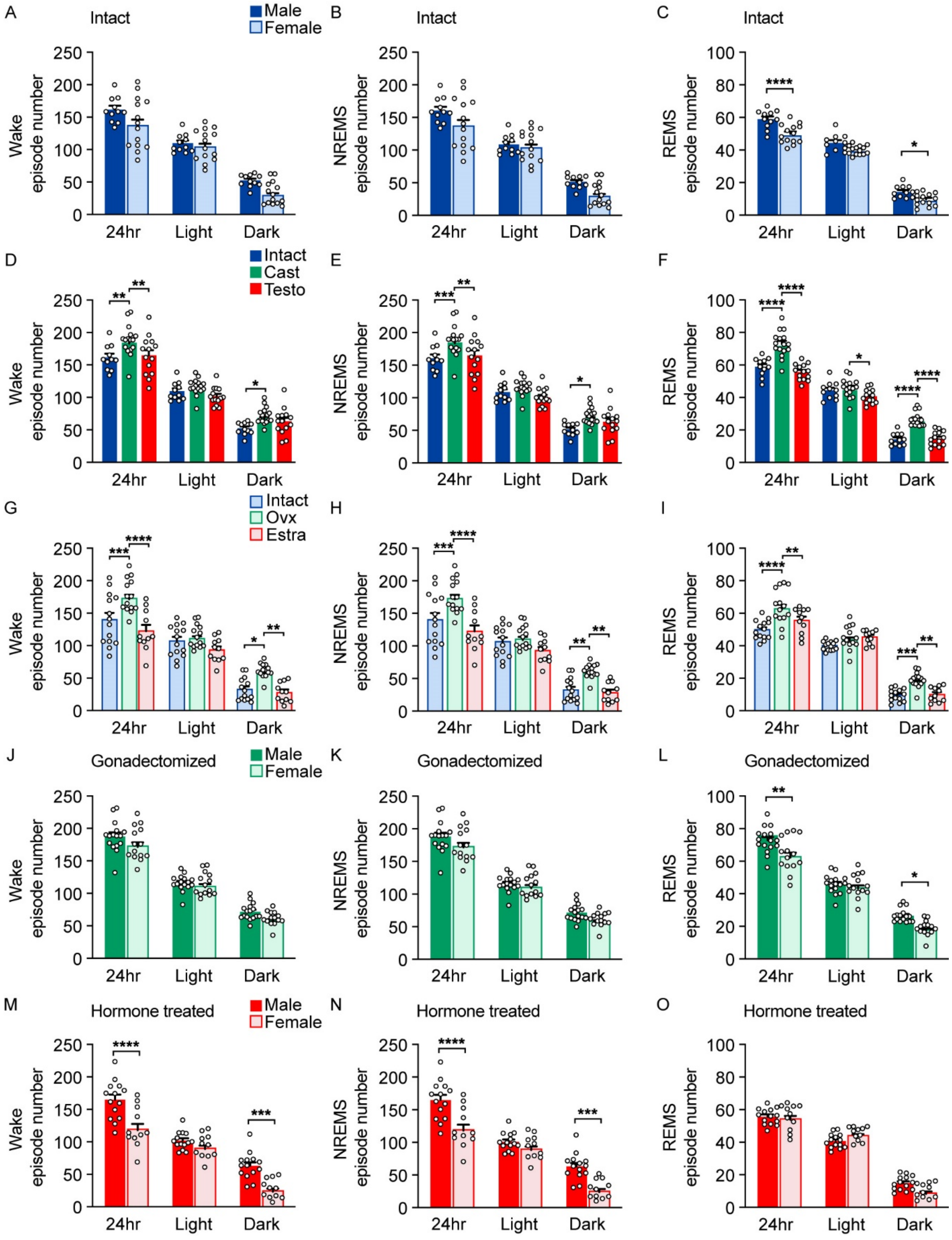

Supplementary Figure 2. Episode numbers of intact, gonadectomized, and hormone-supplemented mice.

(A-C) Episode numbers of wakefulness (A), NREMS (B), and REMS (C) of intact male and female mice. 11 male mice and 14 female mice. Two-way ANOVA followed by Sidak's test. (D-F) Episode numbers of wakefulness (D), NREMS (E), and REMS (F) of male mice. Two-way ANOVA followed by Sidak's test. (G-I) Episode numbers of wakefulness (G), NREMS (H), and REMS (I) of female mice. Two-way ANOVA followed by Sidak's test. (J-L) Episode numbers of wakefulness (J), NREMS (K), and REMS (L) of gonadectomized male and female mice. 16 male mice and 14 female mice. Two-way ANOVA followed by Sidak's test. (M-O) Episode numbers of wakefulness (M), NREMS (N), and REMS (O) of gonadal hormone-supplemented gonadectomized male and female mice. 14 male mice and 11 female mice. Two-way ANOVA followed by Sidak's test. *$P < 0.05$; **$P < 0.01$; ***$P < 0.001$ ****$P < 0.0001$.
